# No Protective Effects of Hair Cells or Supporting Cells in Ototoxically Deafened Guinea Pigs upon Administration of BDNF

**DOI:** 10.3390/brainsci12010002

**Published:** 2021-12-21

**Authors:** Annamaria Tisi, Jochebed Rovers, Henk A. Vink, Dyan Ramekers, Rita Maccarone, Huib Versnel

**Affiliations:** 1 Department of Applied Clinical Sciences and Biotechnology, University of L′Aquila, 67100 L′Aquila, Italy; annamaria.tisi@univaq.it (A.T.); rita.maccarone@univaq.it (R.M.); 2 Department of Otorhinolaryngology and Head & Neck Surgery, University Medical Center Utrecht, Utrecht University, Room G.02.531, P.O. Box 85500, 3508 GA Utrecht, The Netherlands; j.rovers2@students.uu.nl (J.R.); h.a.vink@umcutrecht.nl (H.A.V.); d.ramekers@umcutrecht.nl (D.R.); 3 UMC Utrecht Brain Center, Utrecht University, Universiteitsweg 100, 3584 CG Utrecht, The Netherlands

**Keywords:** organ of Corti, BDNF, hearing loss, hair cells, supporting cells

## Abstract

We investigated whether treatment with brain-derived neurotrophic factor (BDNF), which is known to protect spiral ganglion cells (SGCs), could also protect hair cells (HCs) and supporting cells (SCs) in the organ of Corti of a guinea pig model of sensorineural hearing loss. Hearing loss was induced by administration of kanamycin/furosemide and two BDNF treatments were performed: (1) by gelatin sponge (BDNF-GS) with acute cochlear implantation (CI), and (2) through a mini-osmotic pump (BDNF-OP) with chronic CI. Outer HCs (OHCs), inner HCs (IHCs), Border, Phalangeal, Pillar, Deiters’, and Hensen’s cells were counted. The BDNF-GS cochleas had significantly fewer OHCs compared to the untreated ones, while the IHC and SC numbers did not differ between treated and untreated cochleas. The BDNF-OP group showed similar cell numbers to the untreated group. SGC packing density was not correlated with the total number of SCs for either BDNF group. Our data suggest that: (1) BDNF does not prevent cell death in the organ of Corti, and that the protection of SGCs could result from a direct targeting by BDNF; (2) BDNF might induce a different function/activity of the remaining cells in the organ of Corti (independently from cell number).

## 1. Introduction

The organ of Corti is responsible for the mechano-electrical transduction of sounds and it is a complex epithelium constituted by several cell types located on the basilar membrane of the cochlea. The mechanosensory hair cells (HCs) of the organ of Corti allow the conversion of sound-induced vibrations into electrochemical signals [1]. They are organized in three rows of outer HCs (OHCs) and one row of inner HCs (IHCs), surrounded by non-sensory supporting cells (SCs) that play a major role in the maintenance of structural and functional properties of the sensory epithelium [1,2,3]. Five different types of SCs, organized in rows along the organ of Corti, have been identified based on morphological and functional differences and differences in expression pattern: (1) border cells, (2) inner phalangeal cells, (3) pillar cells, (4) Deiters’ cells, and (5) Hensen’s cells [2]. A gradient exists in the structure and function of HCs and SCs from base to apex in the cochlea [4], in accordance with its tonotopic organization, with the base responsible for transduction of high sound frequencies and the apex for that of low frequencies [5]. A recent study, based on single-cell RNA sequencing, revealed that gene expression, and therefore the function of the cells in the organ of Corti, also follows a gradient from base to apex [4].

Hearing loss represents the fourth most significant cause of disability in the world according to the Global Burden of Disease Study, and the number of affected patients is expected to increase in the next several years [6,7]. The exposure to several damaging stimuli, such as ototoxic drugs and excessive noise, as well as genetic predisposition and aging can induce degeneration of the organ of Corti, leading to irreversible hearing loss and deafness [8]. In particular, in sensorineural hearing loss the mechanosensory HCs are often the primary cells to degenerate, and their death is often followed by progressive degeneration of afferent spiral ganglion cells (SGCs) [9,10,11]. This is a potential problem for the beneficial outcome of cochlear implants (CIs). CIs are surgically implanted electrical stimulators connected to electrode arrays that are inserted into the scala tympani of the cochlea which directly stimulate SGCs and allow sound perception, thus bypassing the damaged organ of Corti [12]. A significant correlation between the performance of CIs and SGC survival has been previously demonstrated [13]. Therefore, progressive SGC death, which occurs as a consequence of the degeneration of the organ of Corti, would lead to reduced efficacy of CIs. For this reason, protective strategies to stop or delay SGC degeneration are needed, and several studies are ongoing in order to achieve this objective and allow prolonged hearing performance in CI users [14,15,16]. More attention has also been drawn to the role of SCs in the organ of Corti in pathological conditions, since the death of HCs is also accompanied by prominent structural alterations of the organ of Corti involving the SCs, and resulting in their degeneration as well [17,18,19,20,21]. In the most advanced stages of the degeneration process, the organ of Corti is then replaced by squamous and cuboidal cells, which constitute a “flat epithelium” [20]. It has been suggested that the remaining non-sensory cells in the deaf cochleas could be used for enhancing SGC survival and function, improving the beneficial effects of CIs. For instance, these cells may be engineered in order to express survival and growth factors to prevent SGC death [22,23] or to induce the regeneration of hair cells [24,25]. Interestingly, it has been shown that the SCs present in the apex of the cochlea show a higher expression of proliferation and differentiation genes [4]. This is particularly important because, in addition to their traditional “supporting” role, in recent years SCs have also been studied as a source to regenerate HCs after damage. First attempts by researchers in the field already showed that the regeneration of HCs from SCs in the mammalian cochlea is actually possible through the targeting of specific signalling pathways [24]. Hence, the protection of SCs, in addition to that of the primary sensory cells, represents a challenging objective to be achieved for therapeutic purposes. Nevertheless, to date effective treatments to prevent cell death in the organ of Corti, or to restore its cells after degeneration, are not available yet, and there is increasing interest in the development of new therapies.

In this context, the administration of neurotrophins represents a new promising therapeutic approach. The neurotrophins form a subclass of neurotrophic factors, a family of proteins, which have been demonstrated to have an essential role in the development, survival, plasticity and protection of the peripheral and central nervous system [26,27]. BDNF is the most abundant neurotrophin of the brain and is essential for inner ear development and survival [28,29]. Notably, recombinant human BDNF administration was effective in preventing SGC death following hair cell degeneration in vivo [15,30,31,32], and therefore represents a promising therapeutic strategy for the treatment of sensorineural hearing loss. In particular, it could be used to further improve the hearing outcomes of patients with a CI by stimulating and sustaining neural health after implantation through the administration of exogenous BDNF. Also, it is considered for treatment of synaptopathy [33,34,35]. In the adult inner ear, the expression of BDNF by HCs and SCs has been demonstrated to be fundamental for the survival of SGCs [29]. Conversely, little is known about the protection of the organ of Corti by neurotrophins, and the identification of NF receptors in both HCs and SCs [36,37] supports the possibility to develop targeted NF-based therapies for the organ of Corti. Moreover, strategies for local drug delivery to the inner ear are of increasing interest among scientists, especially due to the small amount of drug reaching the cochlea after systemic administration, and due the possible systemic side effects [38].

Therefore, in the present study we investigated the effects of recombinant human BDNF on HCs and SCs of the organ of Corti in a model of kanamycin-induced severe sensorineural hearing loss. We re-analyzed data from our previously published experiments in which BDNF was administered via two different routes, those being (1) intracochlear administration based on gelatine sponge [15], and (2) mini-osmotic pumps [32]. We also compared the number of SCs with the survival of SGCs in both of the experimental conditions, in order to investigate whether SCs may protect SGCs even in the absence of HCs.

## 2. Materials and Methods

### 2.1. Animals and Experimental Design

The present study was conducted on cochleas derived from previous studies conducted in the UMC Utrecht [15,32].

Twenty-four young adult albino guinea pigs (Dunkin Hartley; Envigo, Horst, the Netherlands) were kept under standard housing conditions throughout the experiment (food and water ad libitum; lights on between 7:00 a.m. and 7:00 p.m.; temperature 21 °C; humidity 60%). In both of the studies, the animals (except for the normal-hearing [NH] controls) were ototoxically deafened at the start of the experiment, and BDNF was administered through gelatin sponge or osmotic pump to the right cochleas followed by acute or chronic implantation, respectively. At the end of each study, cochlear histological analyses were performed in order to investigate the number of HCs and SCs.

**Figure 1:**
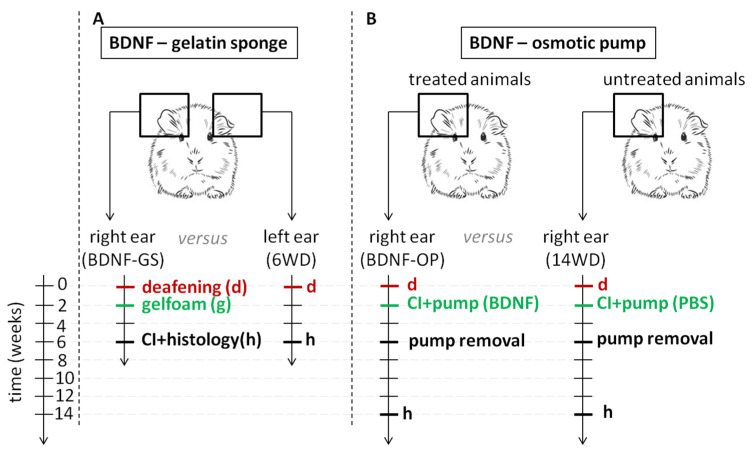
Schematic illustration of the experimental groups. (**A**) Study A was conducted on deafened guinea pigs which received the BDNF-gelatin sponge treatment in the right ears two weeks after deafening, while the contralateral left ear was used as internal (within-subject) negative control; animals received a cochlear implant (CI) in their right ears, six weeks after deafening for acute electrophysiological recordings, and then were euthanized and the cochleas collected for histological analysis. (**B**) Study B was conducted on deafened guinea pigs which received a CI connected to a BDNF-filled osmotic pump in the right ears two weeks after deafening; the osmotic pump was removed after four weeks and the animals were euthanized eight weeks thereafter; the right BDNF-treated ears were compared with the right ears of guinea pigs which underwent the same procedures, but with the osmotic pump filled with vehicle (PBS). For both studies, normal hearing (NH) animals without any treatments were used as baseline. d: deafening, g: gelatin sponge, CI: cochlear implant, h: histology.

Specifically, for BDNF treatment with gelatin sponge (Figure 1, study A), the right cochleas were treated with a piece of gelatin sponge soaked in BDNF two weeks after deafening (labeled BDNF-GS), while the left cochleas were used as internal negative controls (labeled six weeks deaf, 6WD) (N = 11). Four weeks after treatment, the animals received a CI (acute implantation) for electrophysiological recordings immediately prior to euthanasia, and the cochleas were processed for histological analysis [15].

For BDNF administration through mini-osmotic pumps (Figure 1, Study B), all animals received a CI (chronic implantation) in the right ear two weeks after deafening. The electrode array of the CI was combined with a cannula, connected to an osmotic pump inserted subcutaneously anterior to the left shoulder of the animals. The osmotic pump was filled with BDNF (labeled BDNF-OP; N = 7) or vehicle (Phosphate Buffered Saline-PBS). Since the chronic implantation could influence cell survival, we chose not to include the contralateral ears for those animals and the treated cochleas were compared with those of the PBS-treated animals (labeled 14 weeks deaf, 14WD; N = 6). Four weeks after implantation, the osmotic pumps with the cannula were surgically removed to stop the treatment, and eight weeks after treatment cessation all animals were sacrificed and histology was obtained [32].

In both the experimental paradigms, BDNF treatment started two weeks after deafening for the following reasons: (i) The original experiments were performed in order to investigate the protective effects of BDNF on the auditory nerve; therefore, a condition in which the nerve was degenerating was needed; (ii) It is a realistic condition from a clinical point of view; and (iii) There is a more stable hearing loss condition than earlier times after deafening [9].

An overview of the timeline and experimental groups involved in the study is shown in Figure 1.

### 2.2. Deafening Procedure and Treatment Administration

Surgical techniques and experimental procedures for both deafening and treatment administration were conducted according to previously published protocols [15,32]. Anesthesia for both procedures was induced with 40 mg/kg ketamine i.m. (Narketan; Vetoquinol B.V., Breda, The Netherlands) and 0.25 mg/kg dexmedetomidine i.m. (Dexdomitor; Vetoquinol B.V.). Prior to the deafening surgery, normal hearing was verified with click-evoked auditory brainstem responses (ABRs). When normal hearing was confirmed, deafening was performed by systemic delivery of 400 mg/kg kanamycin subcutaneously (Sigma-Aldrich, St. Louis, MO, USA) and 100 mg/kg furosemide i.v. (Centrafarm, Etten-Leur, the Netherlands). Two weeks after deafening, the animals were again anesthetized in order to assess the extent of hearing loss through ABR recordings, and for the administration of BDNF to the right cochleas. ABR recordings were also performed at the end of each experiment, and no differences were observed from two to 14 weeks after deafening [32].

For BDNF administration by means of gelatin sponge, the right bulla was exposed via retro-auricular approach. A small hole was drilled into the bulla to visualize the cochlear basal turn and round window niche. A ~1 mm^3^ piece of gelatin sponge (Spongostan Dental; Ethicon, Somerville, NJ, USA) soaked in BDNF (6.67 mg/mL; PeproTech, Rocky Hill, NJ, USA) was placed into the round window niche, touching the perforated round-window membrane. The animals received the non-ototoxic antibiotic enrofloxacin (Baytril; Bayer AG, Leverkusen, Germany; 5 mg/kg) and carprofen (Carporal; AST Farma, Oudewater, the Netherlands; 4 mg/kg) at the end of each surgery. Four weeks after treatment administration, the animals received CIs for electrophysiological recordings, and were subsequently euthanized and the cochleas collected for subsequent analysis [15].

For BDNF delivery by means of mini-osmotic pumps, the array/cannula was inserted via a cochleostomy in the scala tympani of the basal turn of the cochlea for approximately 4 mm following a retro-auricular approach two weeks after the start of the experiment. The electrode array of the CI was combined with a cannula connected to an osmotic pump. The osmotic pump was inserted subcutaneously anterior to the left shoulder of the animals. Forty hours before surgery, the osmotic pump was filled with PBS containing 1% guinea pig serum (Sigma-Aldrich) for the NH and deaf animals, without treatment. For the deaf BDNF-treated animals, 100 µg/mL BDNF (Peprotech) was added. The pump’s flow rate was 0.25 µL/hour, resulting in a total infusion dosage of approximately 17 µg per animal after twenty-eight days. Four weeks after implantation, the osmotic pumps with the cannula were surgically removed to stop the treatment. Eight weeks after treatment cessation all animals were sacrificed and histology was obtained [32].

### 2.3. Tissue Processing and Histology

For histological analysis, the cochleas were fixed by an intra-labyrinthine infusion with a fixative solution containing 3% glutaraldehyde, 2% formaldehyde, 1% acrolein and 2.5% dimethyl sulfoxide (DMSO) in a 0.08 M sodium cacodylate buffer. The cochleas were then decalcified, post-fixated and embedded in Spurr’s low-viscosity resin. Staining was performed using 1% methylene blue, 1% azur B and 1% borax in distilled water. The cochleas were subsequently divided into two halves along a standardized midmodiolar plane, then re-embedded in fresh resin, and cut to obtain semithin (1 μm) sections. More details about this procedure are reported in Kroon et al. (2017) [39]. The images were acquired by using a Leica DC300F digital camera mounted on a Leica DMRA light microscope with a 40× oil immersion objective (Leica Microsystems GmbH, Wetzlar, Germany).

### 2.4. Cell Count Analysis

**Figure 2:**
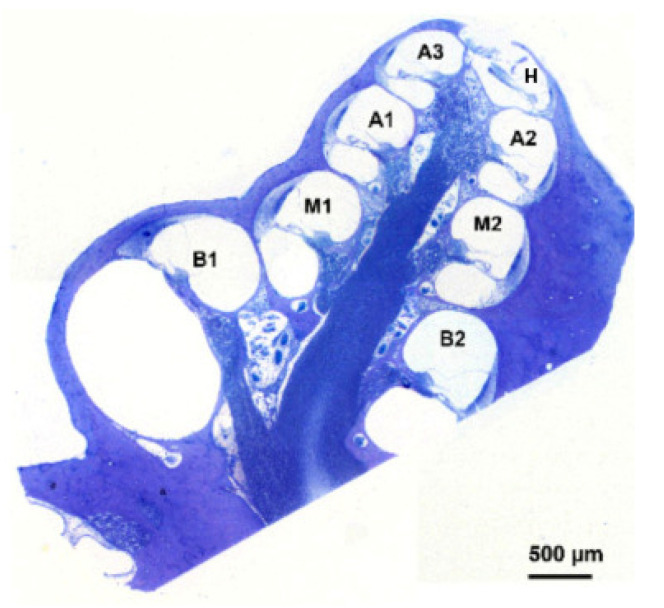
Representative cochlear cross-section. The image is representative of a cochlear midmodiolar section in which all the cochlear locations have been indicated by appropriate lettering: B1, B2, M1, M2, A1, A2, A3, H. The image has been modified from Vink et al., 2020 [15].

Assessment of cell survival was conducted on the acquired images of the organ of Corti from basal to apical semiturns and in the helicotrema (H): B1, B2, M1, M2, A1, A2, A3, H (Figure 2). B2 was excluded from statistical analysis because the organ of Corti was often missing due to processing of the cochlear sections for axonal analysis [39]. In addition to that of HCs, the presence of five types of supporting cells (SCs) was determined: border, inner phalangeal, pillar, Deiters’ and Hensen’s. In order to create a standardized method for cell counting, specific criteria, based on morphological aspects of the cells, were established as reported in Table 1. The average number of cells for each cytotype determined in NH guinea pigs was used to normalize the cell count, and data were therefore expressed as percentage of these NH controls. Specific SC numbers of NH animals for each cochlear location is reported in Section 2.5.

Cell count results of SCs were correlated with SGC packing density of the same animals for each turn: B (B1–B2), M (M1–M2), A (A1, A2, A3). Also, the overall SCs number was correlated with SGC packing density in individual animals. SGC data were obtained from previous analyses [15,32].

### 2.5. Determination of Normalization Factors

**Figure 3:**
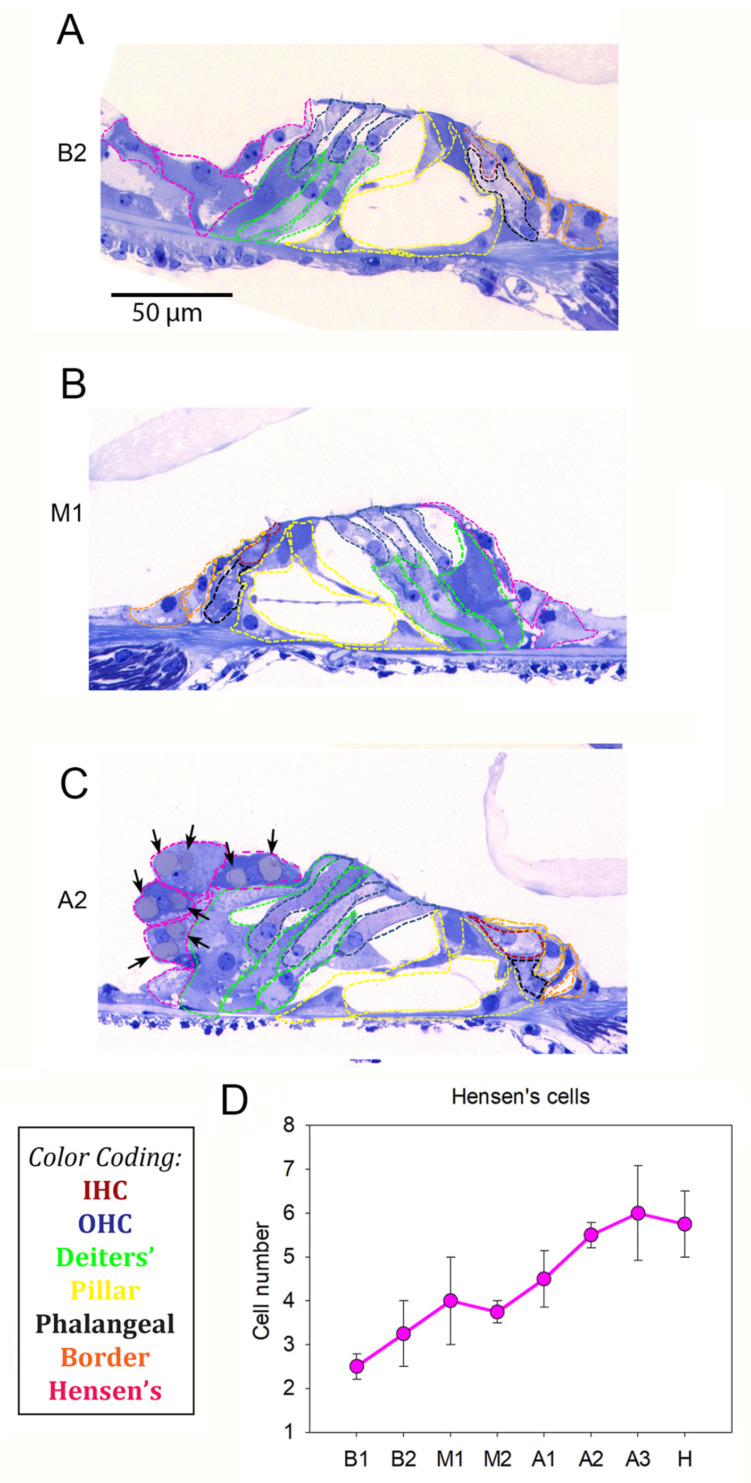
The organ of Corti in normal-hearing guinea pigs. Representative images of a (**A**) B2, (**B**) M1 and (**C**) A2 turn from a NH guinea pig cochlea. Hair cells and supporting cells were marked by using the color coding reported in the black frame: inner hair cells (red), outer hair cells (blue), Deiters’ (green), pillar (yellow), phalangeal (black), border (orange) and Hensen’s (pink). The black arrows indicate the vacuoles present in the Hensen’s cells of the apical turn. (**D**) Hensen’s cell count along the cochlear turns of NH guinea pigs shows an increase from base to apex, visualized as mean ± SEM, as also clearly visible from the images.

As a first step of the study, we focused on NH guinea pig cochleas and analyzed the number of SCs for each cochlear turn in order to set up the normalization numbers to be used for subsequent analysis. To better identify the cell types present in the organ of Corti, a color coding cell signature was performed on the images to highlight the cell edges (Figure 3A–C). HCs were included in the analysis. SC numbers did not differ between the cochlear turns for pillar, Deiters’ and phalangeal cells. Small differences were found in the number of border cells that did not statistically vary between the cochlear locations (not shown). On this basis, the normalization values were the same for each cochlear turn: border (three cells/turn = 100%), phalangeal (one cell/turn = 100%), pillar (two cells/turn = 100%), Deiters’ (three cells/turn = 100%). Conversely, Hensen’s cell number increased from base to apex (Figure 3D). On this basis, different normalization factors were applied depending on the semiturn: B1,B2: 3 cells/semiturn = 100%; M1,M2: 4 cells/semiturn = 100%; A1: 5 cells/semiturn = 100%; A2,A3,H: 6 cells/semiturn = 100%. The increase in the number of Hensen’s cells towards the apex was accompanied by a change in morphology, with the apical Hensen’s cells rich in lipid droplets (Figure 3C, black arrows), as also reported in the literature [40].

### 2.6. Statistical Analysis

For statistical analysis of cell count in the organ of Corti, the non-parametric Mann Whitney and Wilcoxon tests were applied. The Mann Whitney test was performed for the analysis of SGC packing density. Linear regression analysis was performed on SGC packing density (dependent) and SC number (independent). The statistical analysis was conducted by using the SigmaPlot 12.0 software (Systat Software, San Jose, CA, USA). Data are shown as mean ± standard error of mean.

## 3. Results

### 3.1. Sensory and Non-Sensory Cell Counts in Animals Treated with BDNF via Gelatin Sponge

**Figure 4:**
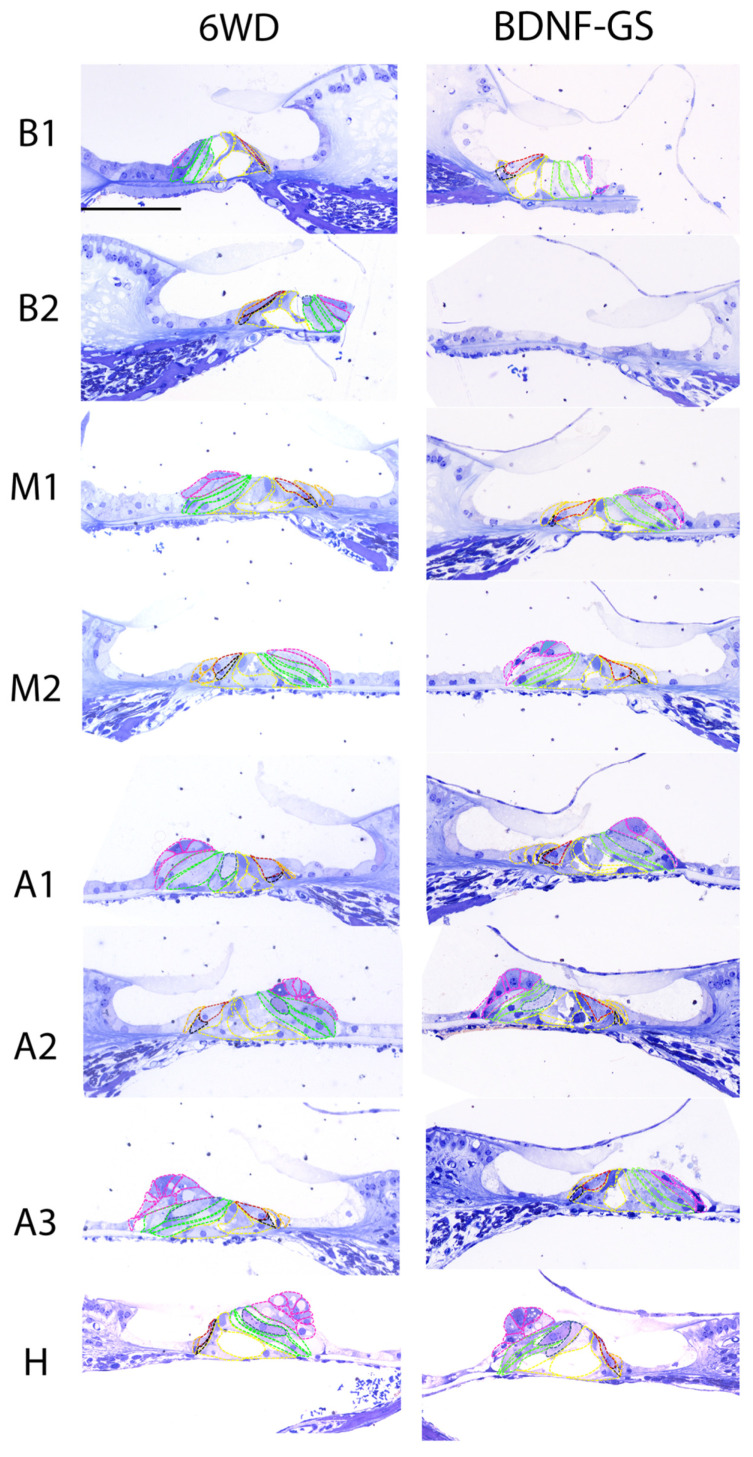
Representative images of the organ of Corti from BDNF-treated animals through gelatin sponge. Microscope images are representative of the organ of Corti of untreated (six weeks deaf: 6WD) (**left**) and BDNF-GS-treated cochleas (BDNF-GS) (**right**) of all cochlear locations: B1, B2, M1, M2, A1, A2, A3, H. Hair cells and supporting cells were marked by using a color coding: outer hair cells (blue), inner hair cells (red), border (orange), phalangeal (black), pillar (yellow), Deiters’ (green), and Hensen’s (pink). Scale bar: 50 µm.

In the first experimental paradigm of the present study, we analyzed the number of sensory and non-sensory cells in both the BDNF-treated (BDNF-GS) cochleas with acute implantation and the contralateral untreated cochleas (6WD) via gelatin sponge [15]. Six weeks after deafening, the organ of Corti appeared to be clearly damaged in both BDNF-GS and 6WD cochleas (Figure 4). The organ of Corti displayed a collapsed structure with loss of the tunnel of Corti and alterations of the typical morphology of each cell type compared to normal-hearing animals. Of note, in the apical turns, a reduced number of vacuoles in the Hensen’s cells was observed. A more severe degeneration was observed in the basal turns compared to the apical ones, which is in agreement with the literature [9]. Figure 4 shows representative images of the organ of Corti from treated and untreated ears for each cochlear location.

**Figure 5:**
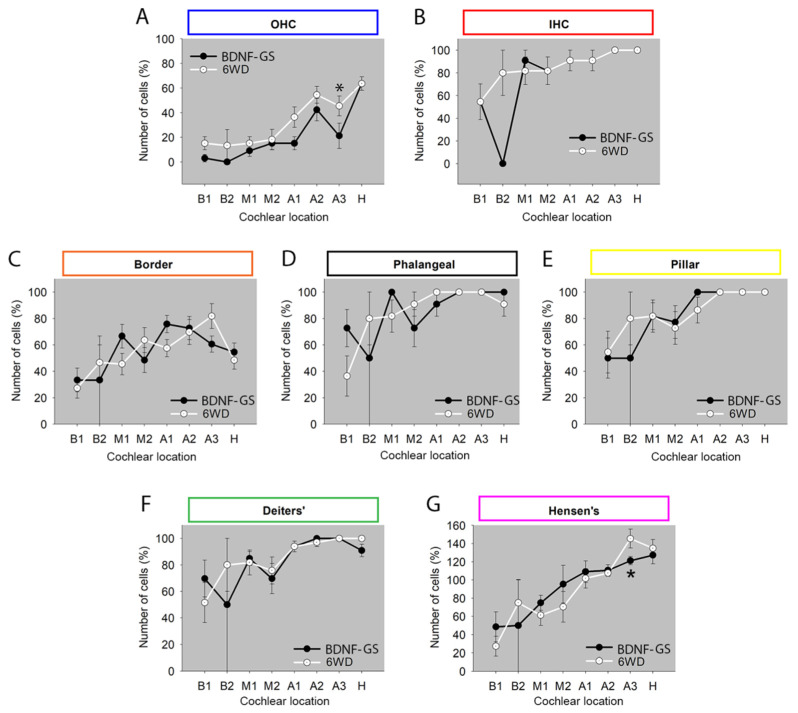
Cell count of sensory and non-sensory cells in the organ of Corti of BDNF-GS-treated ears over all cochlear locations. Cell count of (**A**) outer hair cells (OHC), (**B**) inner hair cells (IHC), (**C**) border, (**D**) phalangeal, (**E**) pillar, (**F**) Deiters’, (**G**) Hensen’s cells. Graphs are shown as mean ± SEM (n = 11) and show cell count of each cell type for each cochlear location of BDNF-GS and untreated ears (6WD). * *p* < 0.05, statistical analysis: Mann Whitney. Number of cells is expressed as percentage of NH (100%).

Comparison of the number of OHCs revealed that the BDNF-GS cochleas presented fewer cells at the A3 location compared to the untreated ones, with a statistically significant difference (not corrected for multiple testing; *p* = 0.041) (Figure 5A). In the same cochlear location, the number of Hensen’s cells was also significantly lower in the treated group compared to the untreated one (*p* = 0.028). Conversely, the IHC number averaged by the experimental group did not vary between the treated and untreated ears for each cochlear turn (Figure 5B). Furthermore, the analysis of SCs demonstrated very similar cell numbers for each supporting cell type for each cochlear location (*p* > 0.07) (Figure 5C–G).

**Figure 6:**
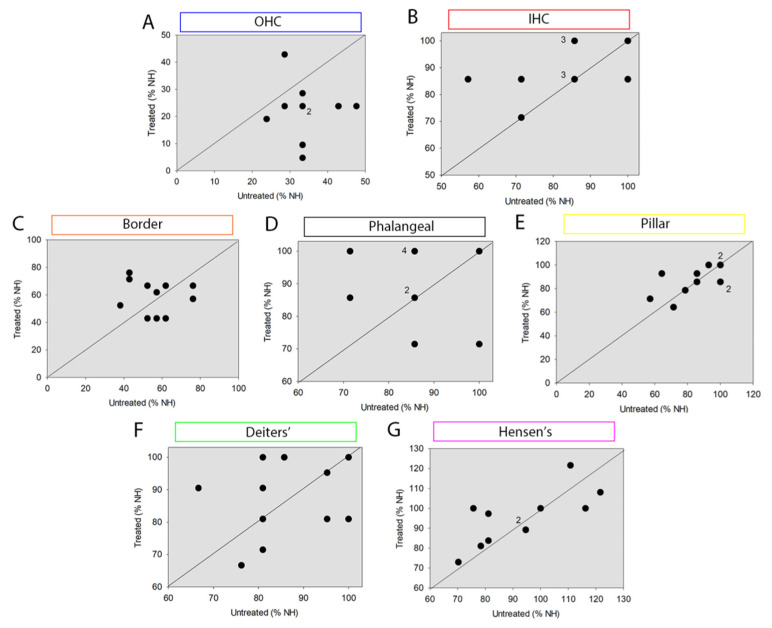
Scatter plot of the number of sensory and non-sensory cells of BDNF-GS and untreated (6WD) cochleas in individual animals. Cell count of (**A**) outer hair cells (OHC), (**B**) inner hair cells (IHC), (**C**) border, (**D**) phalangeal, (**E**) pillar, (**F**) Deiters’, (**G**) Hensen’s cells. The scatter plots show the total number of SCs of treated versus untreated cochleas of individual animals. Number of cells is expressed as percentage of NH (100%). Incidentally overlapping data points are indicated by the adjacent number, indicating the number of overlapping data points. Diagonal lines represent y = x.

BDNF treatment in the right cochlea only allowed us to compare contralateral ears of individual animals, which gave us a wider overview of the protection/degeneration of the organ of Corti in our experimental conditions. The difference in OHC number was even more evident in the scatter plot obtained comparing the total number of OHC over all cochlear locations for individual animals (Figure 6A). Accordingly, the overall difference of OHC number between treated and untreated ears of individual animals was statistically significant (on average 24.2% in treated vs. 35.5% in untreated cochleas, *p* = 0.019). In contrast to OHCs, no differences were found between treated and untreated cochleas of individual animals with respect to either the total number of IHCs (average treated 87% vs. untreated 85.7%, Figure 6B) or all the supporting cell types (Border: average treated 58.9% vs. untreated 56.3%, Figure 6C; Phalangeal: average treated 90.9% vs. untreated 85.7%, Figure 6D; Pillar: average treated 87% vs. untreated 85.1%, Figure 6E; Deiters’: average treated 87% vs. untreated 85.7%, Figure 6F; Hensen’s: average treated 94.8% vs. untreated 93.1%, Figure 6G) over all cochlear locations (*p* > 0.74)(Figure 6C–G).

### 3.2. Sensory and Non-Sensory Cell Counts in Animals Treated via Osmotic Pump

**Figure 7:**
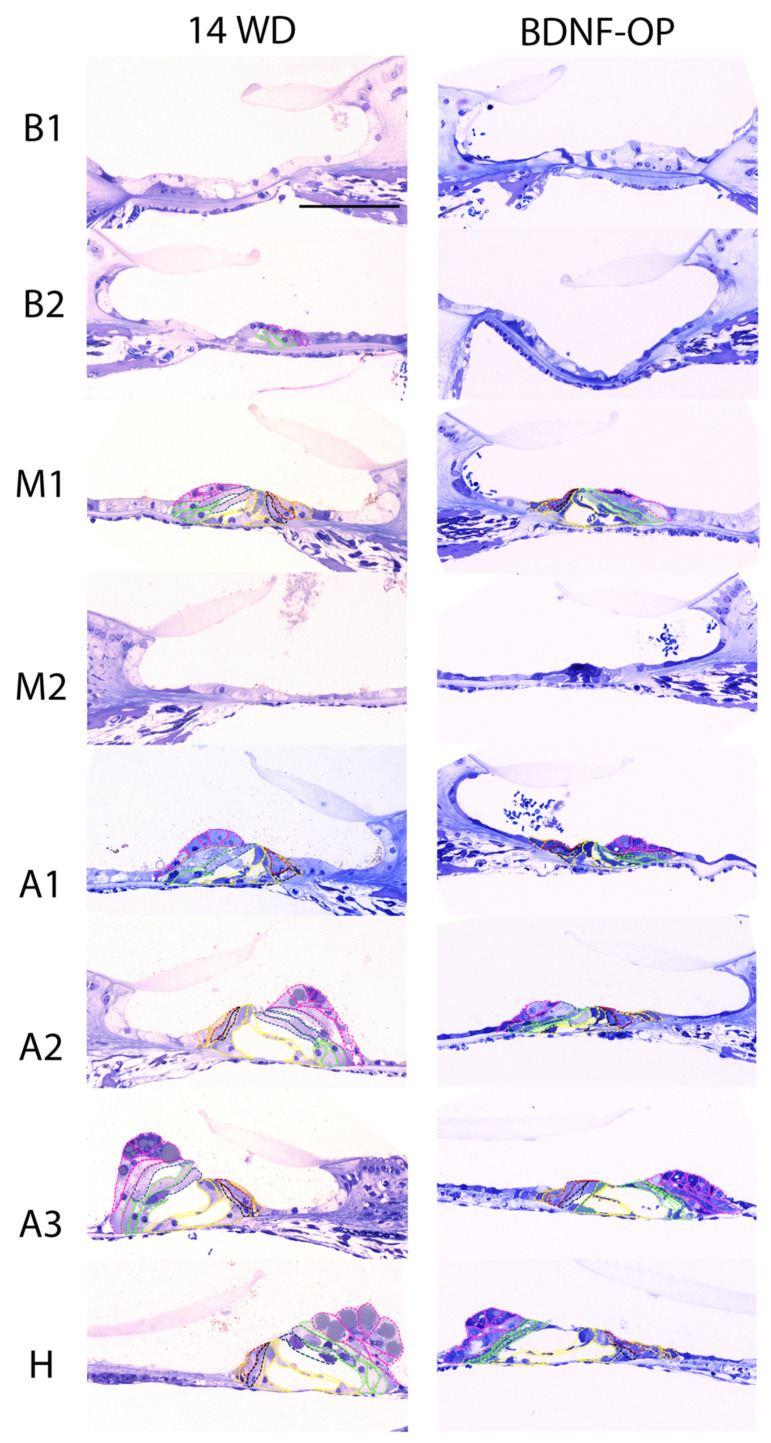
Representative images of the organ of Corti from BDNF-treated animals through mini-osmotic pump (BDNF-OP). Microscope pictures are representative of the organ of Corti of untreated (fourteen weeks deaf: 14WD) (**left**) and BDNF-OP cochleas (**right**) of all cochlear locations: B1, B2, M1, M2, A1, A2, A3, H. Hair cells and supporting cells were identified by using a color coding: outer hair cells (blue), inner hair cells (red), border (orange), phalangeal (black), pillar (yellow), Deiters’ (green), and Hensen’s (pink). Scale bar: 50 µm.

**Figure 8:**
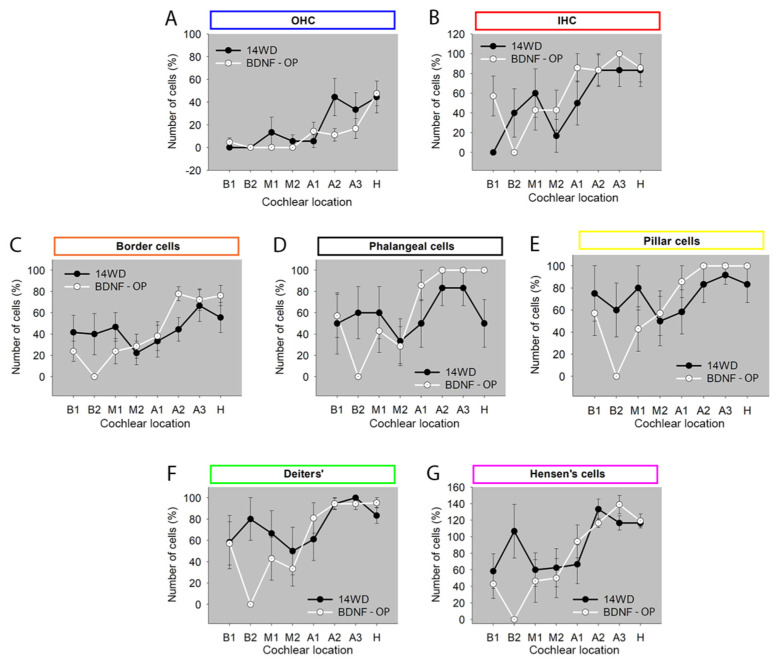
Cell count of sensory and non-sensory cells in the organ of Corti of BDNF-OP ears over all cochlear locations. Cell count of (**A**) outer hair cells (OHC), (**B**) inner hair cells (IHC), (**C**) border, (**D**) phalangeal, (**E**) pillar, (**F**) Deiters’, (**G**) Hensen’s cells. Graphs are shown as mean ± SEM (14WD: n = 6; BDNF-OP: n = 7) and show cell count of each cell type for each cochlear location of BDNF-treated (BDNF-OP) and PBS-treated ears (14WD). Number of cells is expressed as percentage of NH (100%).

In the second experimental paradigm of the present study, we analyzed the number of sensory and non-sensory cells of BDNF-treated animals (BDNF-OP) and PBS-treated animals (14WD) through mini-osmotic pumps and with chronic implantation [32]. The analysis was performed comparing the number of cells of BDNF-OP with the 14WD animals, since several processes triggered by the chronic implantation could have influenced the outcome of the experiment. Fourteen weeks after deafening, the organ of Corti appeared clearly damaged in both BDNF-OP and 14WD cochleas (Figure 7). As for the BDNF-gelatin sponge study, the organ of Corti displayed a collapsed structure in both treated and untreated animals compared to normal-hearing ones. Figure 7 shows some representative images of the organ of Corti from BDNF-OP and untreated ears for each cochlear location. Accordingly, comparison of the number of sensory cells (OHC: average treated 12.9% vs. untreated 20.6%, Figure 8A; IHC: average treated 67.3% vs. untreated 52.4%, Figure 8B) and non-sensory cells (Border: average treated 45.6% vs. untreated 41.3%, Figure 8C; Phalangeal: average treated 69.4% vs. untreated 54.8%, Figure 8D; Pillar: average treated 73.5% vs. untreated 69%, Figure 8E; Deiters’: average treated 67.3% vs. untreated 69%, Figure 8F; Hensen’s: average treated 81.5% vs. untreated 83.7%, Figure 8G) showed very similar cell numbers between BDNF-OP and 14WD cochleas from base to helicotrema. Hence, no statistically significant differences were found between the two experimental groups (*p* > 0.13).

**Figure 9:**
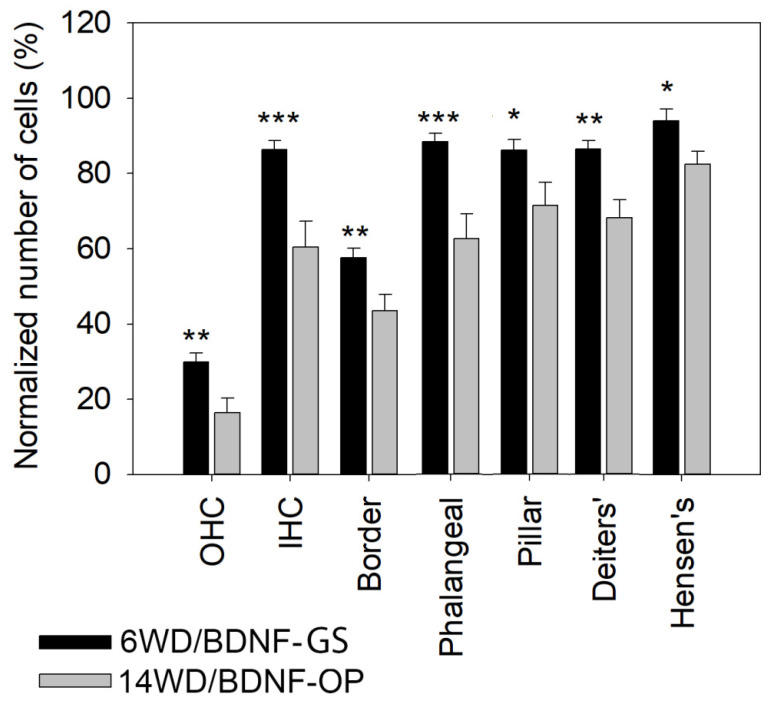
Comparison of HCs and SCs cell numbers between cochleas of six and 14 weeks deaf animals. Treated and untreated cochleas for each time point were merged (6WD/BDNF-GS and 14WD/BDNF-OP). Graphs are shown as mean ± SEM (6WD/BDNF-GS: n = 22; 14WD/BDNF-OP: n = 13) and show total cell count of each cell type over all cochlear locations. Number of cells is normalized as % of NH (100%). Statistical analysis was performed through Mann Whitney test; * *p* < 0.05, ** *p* < 0.01, *** *p* < 0.001.

Since the numbers of hair cells and SCs between treated and untreated cochleas did not differ in both BDNF-GS and BDNF-OP studies, the data were merged in order to analyze the effect of duration of deafness for each cell type of the organ of Corti (from six to fourteen weeks after deafening, that are the BDNF-GS and BDNF-OP studies respectively) (Figure 9). The analysis of specific HCs and SCs number over all cochlear locations showed a significant decrease of all sensory and non-sensory cells from 6 to 14 weeks after deafening (OHC: average 6WD/BDNF-GS 29.9% vs. 14WD/BDNF-OP 16.5%, *p* = 0.007; IHC: average 6WD/BDNF-GS 86.4% vs. 14WD/BDNF-OP 60.4%, *p* < 0.001; Border: 6WD/BDNF-GS 57.6% vs. 14WD/BDNF-OP 43.6%, *p* = 0.008; Phalangeal: 6WD/BDNF-GS 88.3% vs. 14WD/BDNF-OP 62.6%, *p* < 0.001; Pillar: 6WD/BDNF-GS 86% vs. 14WD/BDNF-OP 71.4%, *p* = 0.03; Deiters’: average 6WD/BDNF-GS 86.4% vs. 14WD/BDNF-OP 68.1%, *p* = 0.002; Hensen’s: 6WD/BDNF-GS 93.9% vs. 14WD/BDNF-OP 82.5%, *p* = 0.047). This data suggests a progressive degeneration of the organ of Corti in our experimental model over time.

### 3.3. Correlation between Spiral Ganglion Cell Density and Supporting Cell Number

**Figure 10:**
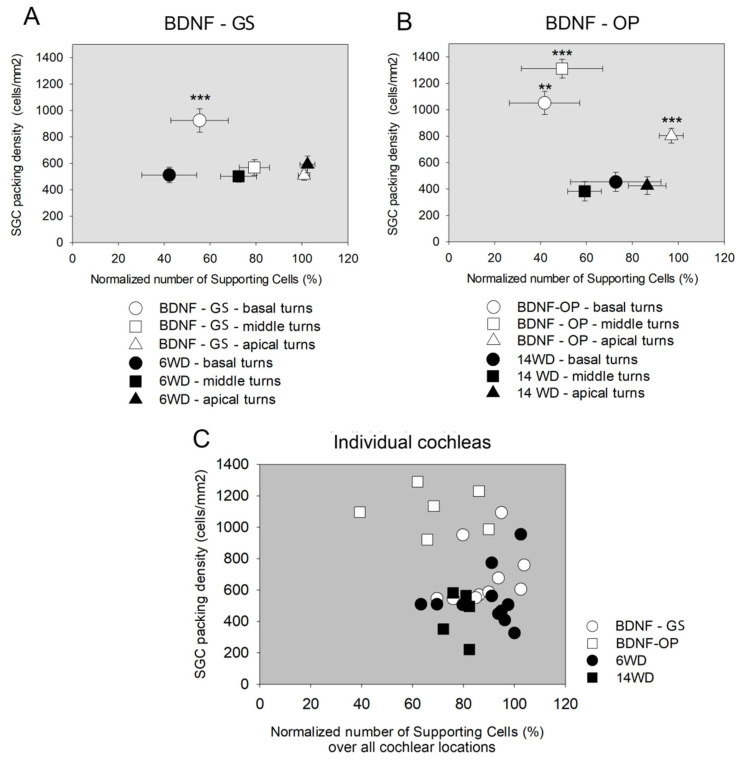
Correlation between spiral ganglion cell (SGC) packing density and SCs number. (**A**) Correlation between the SGC packing density and the total number of supporting cells normalized vs. NH (%) in basal, middle and apical turns of BDNF-GS and 6WD cochleas averaged by treatment group. The graph is expressed as mean ± SEM (SGC packing density: n_BNDF-GS_ = 12, n_6WD_ = 12; Total number of supporting cells: n = 11). (**B**) Correlation between the SGC packing density and the total number of supporting cells normalized vs. NH (%) in basal, middle and apical turns of BDNF-OP and 14WD cochleas averaged by treatment group. The graph is expressed as mean ± SEM (SGC packing density: n_BDNF-OP_ = 7, n_14WD_: = 6; Total number of supporting cells: BDNF: n = 11). Statistical analysis refers to SGC packing density; statistical test: Mann Whitney, ** *p* < 0.01, *** *p* < 0.001 vs. untreated. (**C**) Scatter plot correlating SGC packing density and the overall SCs number for individual ears and animals (BDNF–GS: n = 11; 6WD: n = 11; BDNF-OP: n = 6; 14WD: n = 5) over all cochlear locations (B1, B2, M1, M2, A1, A2, A3).

SCs are gaining increasing interest as important mediators of cell survival of neighboring cells and downstream SGCs [3]. On this basis, we wondered whether a correlation between SGC survival and the number of remaining SCs existed in our experimental conditions. Specifically, we correlated the SGC packing density with the total number of SCs, considering the total amount derived from the sum of all the SC types (Figure 10). The BDNF-GS cochleas showed a significantly increased SGC packing density in the basal turns compared to the untreated cochleas (*p* < 0.001) (Figure 10A), as previously reported [15]. Conversely, the SGC packing density was similar between treated and untreated ears in the middle and apical turns (Figure 10A). However, this data was not associated with any differences in the total SC number, which instead was similar in treated and untreated ears over all cochlear locations (Figure 10A). The BDNF-OP group showed a different scenario, with a significantly increased SGC packing density in the basal, middle, and apical turns compared to the untreated group (Base: *p* < 0.01, Middle and Apex: *p* < 0.001) (Figure 10B). However, as also observed in the BDNF-GS study, no correlation was found between the SGC survival and the number of SCs. Indeed, the total number of SCs was similar for BDNF-OP and vehicle-injected ears in the basal, middle and apical turns (Figure 10B).

Likewise, SGC packing density plotted against the total number of SCs of individual cochleas of both the BDNF studies showed no correlation between the two variables (R^2^ = 0.077, *p* = 0.119) (Figure 10C). We also investigated whether any association existed between SGC packing density and the number of specific subtypes of SCs over all cochlear locations. No statistically significant correlation was found (*p* > 0.13) (data not shown).

## 4. Discussion

In the present study, we investigated the effects of BDNF on the different cytotypes of the organ of Corti in ototoxically deafened guinea pigs. We hypothesized that the organ of Corti could be protected by treatment with exogenous BDNF, and could in turn mediate the protection of afferent SGCs, which are known to be protected by BDNF. Our analysis was focused on the cell number of both sensory and non-sensory cells. Second, we investigated whether a correlation exists between the survival of SGCs and supporting cell number. For this purpose, two different experimental paradigms were taken into account: (1) BDNF administration through gelatin sponge application and with acute cochlear implantation (BDNF-GS), and (2) BDNF administration through a mini-osmotic pump and with a chronic cochlear implantation (BDNF-OP).

### 4.1. Hair Cells and Supporting Cells Are Not Protected by BDNF

The findings of our study indicate that BDNF does not protect the HCs and SCs from degeneration after ototoxic trauma when administered by means of gelatin sponge or through mini-osmotic pumps. However, some considerations regarding this result are in order. First, the treatment was administered two weeks after damage induction and we did not consider any other intermediate time points that would allow one to have a wider overview of degeneration processes and the possible protective effects by both BDNF treatment paradigms. In fact, in principle, the two studies were designed with the aim of investigating the protective effects of BDNF on SGCs [15,32] which degenerate as a consequence of HC death. For this reason, the treatment was administered when a high degree of damage had already occurred in the organ of Corti. Moreover, only one concentration for treatment was tested. It is therefore possible that the effects of BDNF administration may not be appreciable in these experimental conditions, although the organ of Corti was not completely degenerated and a sufficient number of HCs and SCs were still detectable. For instance, an earlier treatment administration after deafening or a higher dosage could result in a more effective protective outcome. Nonetheless, our data are in agreement with the literature, suggesting that BDNF is likely not to be effective in the organ of Corti. For example, a previous study from Shoji and colleagues demonstrated that BDNF administered through osmotic pumps was not able to prevent OHC degeneration in noise-induced deafened guinea pigs even when the treatment started four days before damage and continued until one week after [41]. Likewise, BDNF administered through osmotic pumps was not effective in protecting HCs in guinea pigs deafened by kanamycin, while protective effects of neurotrophin-3 (NT-3) on hair cells were observed in the same study [42].

Moreover, we found that the number of OHCs was reduced in the cochleas treated by gelatin sponge soaked in BDNF compared to the untreated contralateral cochleas, which did not receive the surgery for gelatin sponge administration. This outcome was not observed in the BDNF-OP treated cochleas compared to the untreated ones, which instead underwent the same surgical procedure with osmotic pumps filled with vehicle. This data leads one to hypothesize that the decrease in OHC survival observed in the BDNF-GS study could be a possible negative effect related to the surgical procedure rather than to the exogenous BDNF itself. However, additional investigations would be needed to confirm this hypothesis. It is also important to note that no statistical significance exists after Bonferroni correction for number of comparisons (seven, to be significant *p* = 0.05/7~0.007) for OHC in the BDNF-GS study.

### 4.2. Kanamycin Induces Progressive Degeneration of Hair Cells and Supporting Cells in Guinea Pigs

Progressive degeneration of the organ of Corti involves either sensory and non-sensory cells, and leads to a non-functioning “flat epithelium” in the final stages of the degeneration process [20]. On this basis, here we also investigated the duration of deafness after kanamycin administration and compared the number of surviving cells in the organ of Corti of six (BDNF-GS study) and 14 weeks (BDNF-OP study) deaf guinea pigs. The average percentages of sensory and non-sensory cells in the BDNF-OP study were significantly lower than those of the BDNF-GS study, suggesting that a progressive degeneration of HCs and SCs could continue over time from six to 14 weeks after deafening. However, it should be considered that the BDNF-GS and BDNF-OP studies display different experimental conditions (i.e., chronic cochlear implantation and vehicle administration in the BDNF-OP study), that could have influenced the survival of cells in the organ of Corti. Nonetheless, the notion of progressive degeneration is reasonable. First, according to electrophysiological analysis performed on the same cochleas [15,32], the remaining hair cells are for the most part not functional, indicating a severe state of degeneration already after six weeks (click-evoked ABR threshold shifts of 76 dB in the BDNF-GS study [15], and of 81 dB in the BDNF-OP study [32]). Second, our previous study on kanamycin-induced hearing loss showed progressive HC loss after deafening over time [9]. In addition, here we report for the first time that also the neighboring supporting cells progressively degenerate in our experimental model, making it also a suitable animal model for investigations on degeneration/protection of the supporting cells for sensorineural hearing loss. Finally, we previously demonstrated that short electrode arrays as used in the BDNF-OP and 14WD animals do not damage the HCs, since no differences in HC counts were observed in the implanted cochleas compared to the non-implanted ones, and ABRs indicated only a 10 dB threshold shift at 12 weeks after implantation in NH animals [43]. Furthermore, HCs are generally more vulnerable to ototoxicity than SCs [21,44], and therefore it is expected that CIs do not affect SC survival either. These findings suggest that the increased degeneration observed in the organ of Corti after 14WD is not influenced by CIs, but rather by the progression of the degeneration processes over time upon kanamycin administration. This further supports the reliability of the comparison between the two experimental paradigms of this study.

### 4.3. No Correlation between Protection of the Organ of Corti and SGCs Survival

The protective effect of BDNF on the survival of SGCs was previously demonstrated [15,32]. Both the gelatin sponge and mini-osmotic pumps proved to be effective in protection of SGCs. However, BDNF administered through mini-osmotic pump showed the highest SGCs survival, leading to an increased packing density over all cochlear locations, while BDNF administered via gelatin sponge protected SGCs only in the basal cochlea. We investigated whether the protection of SGCs was associated with the presence of SCs in the organ of Corti [19,20]. However, we found no correlation between the SGC and SC survival, suggesting a direct targeting of SGCs by the BDNF without a role of SCs. Another hypothesis is that the treatment could preserve the function/activity of surviving supporting cells, which could be independent from cell number. Crucially, although the number of cells did not differ between treated and untreated ears, the function and activity of those remaining cells may be different. For instance, BDNF could have promoted the secretion of additional growth and survival factors in the remaining SCs that in turn could influence the health of SGCs [45]. In fact, it is important to note that we conducted only a morphological evaluation of the organ of Corti, and did not take into account any other aspects, such as the molecular events underlying the activity of those cells, that could affect their function. Likewise, BDNF could also have maintained a better functionality of the sensory cells in the organ of Corti, for instance by preventing synaptopathies and maintaining functional ribbon synapses with the afferent fibers [46,47]. In the future, additional studies on the molecular and functional aspects of the remaining cells in the organ of Corti could allow one to get in depth with the most specific mechanisms underlying the crosstalk between the sensory epithelium, the SGCs and BDNF.

## 5. Conclusions

In the present study, we investigated the protection of the organ of Corti by BDNF in an animal model of sensorineural hearing loss. We considered both sensory and non-sensory cells of the organ of Corti which degenerated over time upon kanamycin and furosemide co-administration. All the cytotypes were equally affected in treated and untreated cochleas, and the same result was observed in the two experimental conditions of the study (BDNF-GS and BDNF-OP). Moreover, the protection of SGCs in the BDNF-treated cochleas did not correlate with the number of SCs in the organ of Corti. Taken together, our data indicate that BDNF is likely not to be effective in protecting the organ of Corti from degeneration in ototoxically deafened guinea pigs, and that the protection of SGCs could be a direct targeting of BDNF without a role for SCs or HCs. Alternatively, a different function/activity of the remaining cells in the organ of Corti (which could be independent from cell number) of the BDNF-treated cochleas could mediate SGC protection, and needs to be investigated.

## Figures and Tables

**Table 1:** Criteria for the identification of specific sensory and non-sensory cells of the organ of Corti. For each cell type, morphological aspects for their identification were determined and used to perform the cell count along the cochlear turns.

Cytotype	Morphological Aspects	Inclusion Criteria
IHC	(1) Presence of the cuticular plate/hair bundle; (2) Nucleus at appropriate position; (3) Evident IHC cell edges at the expected position.	Cells included as IHC comply with point 1 and at least one of point 2 and 3.
OHC	(1) Presence of the cuticular plate/hair bundle; (2) Nucleus at appropriate position; (3) Evident OHC cell edges at the expected OHC position.	Cells included as OHC comply with point 1 and at least one of point 2 and 3.
Border	(1) Evident morphological cell edges; (2) Cell counting starts from cells in contact with IHC to the inner sulcus cells (identified as bigger, cuboid and often white cells); (3) Appropriate nucleus position.	Cells included as Border cells comply with at least point 1 and 2.
Phalangeal	(1) Evident morphological cell edges; (2) Position immediately under the IHC or along the IHC side close to Pillar cells; (3) Presence of peculiar white spots; (4) Appropriate nucleus position.	Cells included as Phalangeal comply with at least point 1 and 2.
Pillar	(1) Presence of a triangular cell shape in contact with the basilar membrane; (2) Presence of extensions that widen in the superior part of the organ of Corti.	Cells included as Pillar comply with one of the morphological aspects.
Deiters’	(1) Evident cell edges; (2) Contact with the basilar membrane; (3) Appropriate nucleus position	Cells included as Deiters’ cells comply at least with points 1 and 2.
Hensen’s	(1) Evident morphological cell edges; (2) Presence of a nucleus; (3) Presence of vacuoles.	Cells included as Hensen’s comply with at least point 1 or 2.

## Data Availability

The data presented in this study are available within the article.
